# Immunity to Intracellular *Salmonella* Depends on Surface-associated Antigens

**DOI:** 10.1371/journal.ppat.1002966

**Published:** 2012-10-18

**Authors:** Somedutta Barat, Yvonne Willer, Konstantin Rizos, Beatrice Claudi, Alain Mazé, Anne K. Schemmer, Dennis Kirchhoff, Alexander Schmidt, Neil Burton, Dirk Bumann

**Affiliations:** 1 Focal Area Infection Biology, Biozentrum, University of Basel, Basel, Switzerland; 2 Junior Group “Mucosal Infections”, Hannover Medical School, Hannover, Germany; 3 Department of Molecular Biology, Max-Planck-Institute for Infection Biology, Berlin, Germany; 4 Immunomodulation Group, Deutsches Rheuma-Forschungszentrum Berlin, Berlin, Germany; 5 Proteomics Core Facility, Biozentrum, University of Basel, Basel, Switzerland; Yale University School of Medicine, United States of America

## Abstract

Invasive *Salmonella* infection is an important health problem that is worsening because of rising antimicrobial resistance and changing *Salmonella* serovar spectrum. Novel vaccines with broad serovar coverage are needed, but suitable protective antigens remain largely unknown. Here, we tested 37 broadly conserved *Salmonella* antigens in a mouse typhoid fever model, and identified antigen candidates that conferred partial protection against lethal disease. Antigen properties such as high in vivo abundance or immunodominance in convalescent individuals were not required for protectivity, but all promising antigen candidates were associated with the *Salmonella* surface. Surprisingly, this was not due to superior immunogenicity of surface antigens compared to internal antigens as had been suggested by previous studies and novel findings for CD4 T cell responses to model antigens. Confocal microscopy of infected tissues revealed that many live *Salmonella* resided alone in infected host macrophages with no damaged *Salmonella* releasing internal antigens in their vicinity. In the absence of accessible internal antigens, detection of these infected cells might require CD4 T cell recognition of *Salmonella* surface-associated antigens that could be processed and presented even from intact *Salmonella*. In conclusion, our findings might pave the way for development of an efficacious *Salmonella* vaccine with broad serovar coverage, and suggest a similar crucial role of surface antigens for immunity to both extracellular and intracellular pathogens.

## Author Summary

*Salmonella* infections cause extensive morbidity and mortality worldwide. A vaccine that prevents systemic *Salmonella* infections is urgently needed but suitable antigens remain largely unknown. In this study we identified several antigen candidates that mediated protective immunity to *Salmonella* in a mouse typhoid fever model. Interestingly, all these antigens were associated with the *Salmonella* surface. This suggested that similar antigen properties might be relevant for CD4 T cell dependent immunity to intracellular pathogens like *Salmonella*, as for antibody-dependent immunity to extracellular pathogens. Detailed analysis revealed that *Salmonella* surface antigens were not generally more immunogenic compared to internal antigens. However, internal antigens were inaccessible for CD4 T cell recognition of a substantial number of infected host cells that contained exclusively live intact *Salmonella*. Together, these results might pave the way for development of an efficacious *Salmonella* vaccine, and provide a basis to facilitate antigen identification for *Salmonella* and possibly other intracellular pathogens.

## Introduction

Enteric fever caused by systemic *Salmonella* infection causes tremendous morbidity and mortality worldwide [1]. Current control strategies become increasingly inefficient as a result of increasing antimicrobial resistance [2], [3] and emergence of *Salmonella* serovars that are not covered by currently available safe vaccines [4], [5]. This situation generates an urgent medical need for novel *Salmonella* vaccines with broad serovar coverage.

Early killed whole-cell vaccines containing mixtures of different serovars provide broad protection, but cause unacceptable adverse reactions [1]. As an alternative to whole-cell vaccines, subunit vaccines containing a few defined *Salmonella* components could minimize adverse reactions. Indeed, vaccines containing the capsular polysaccharide Vi antigen provide moderate protection and excellent safety [1]. On the other hand, serovars Paratyphi A and non-typhoidal *Salmonella* (NTS) that cause an increasing number of invasive salmonelloses [6], lack the Vi antigen and are therefore not covered by Vi vaccines [5]. Apart from Vi, few *Salmonella* antigens have been identified, and all of these provide at best moderate levels of protection against challenge infection with virulent *Salmonella* strains in the commonly used mouse typhoid fever model. Moreover, antigens such as flagellin [7] and OmpD [8] are poorly conserved among relevant serovars.

For extracellular pathogens with antibody-mediated immunity, protective antigens must be surface-exposed [9], and this enables an effective strategy for priorization of antigen candidates [9]. Humoral response to surface antigens can also contribute to immunity to intracellular pathogens such as invasive *Salmonella* [10]. Indeed, Vi which induces protective antibody responses in human vaccinees, forms an extracellular capsule around *Salmonella* Typhi [11]. Two additional antigens that confer partial immunity in the mouse typhoid fever model, flagellin [7] and SseB [12], are also part of *Salmonella* surface structures (flagella, translocon complex of a type III secretion system). Furthermore, outer membrane preparations (but not the outer membrane component lipopolysaccharide) have been suggested to mediate protective humoral immune responses against extracellular *Salmonella* bacteremia [13] and attenuated *Salmonella* strains in the mouse model [8], [14]. A number of porins such as OmpC, OmpD, and OmpF are highly abundant in such outer membrane preparations suggesting that they might represent the actual protective antigens [8], [14], [15].

However, immunity to *Salmonella* critically depends also on CD4 T cells [10]. Unfortunately, protective T cell antigens seem to be rare, and priorization of candidates is difficult since relevant antigen properties for CD4 T cell responses remain unclear [9], [16], [17]. One key precondition for protective responses is expression of the respective *Salmonella* antigen during infection [18], and some data suggest that highly abundant antigens might be particularly well recognized by CD4 T cells [12], [19]. Antigen in vivo expression can be deduced from various complementary approaches including screening of promoter trap libraries [20], [21], proteomics [22], serum antibody response [23]–[26], as well as mutant virulence phenotypes.

In addition to antigen expression, antigen immunogenicity could play a major role. Antigen detection by cognate CD4 T cells requires antigen processing and presentation of the resulting small peptides by major histocompatibility (MHC) class II molecules. Peptide sequence properties that are characteristic for well recognized epitopes, can be used for genome-wide prediction of promising antigens [27]. However, a large number of non-protective antigens contain putative high-score epitopes [16], [18], [28] which could compromise the discriminatory power of this approach.

Experimental detection of immune responses to an antigen in convalescent individuals that have survived infection, demonstrates that this antigen was expressed in vivo and could be recognized by the immune system [23]–[25]. Indeed, this approach has been recently shown to facilitate identification of protective *Chlamydia* antigens [29]. On the other hand, many immunodominant antigens in convalescent individuals lack protective efficacy, while a number of protective antigens may induce immune responses below the detection threshold during natural infection [17].

Another antigen property that can affect CD4 T cell responses is antigen localization. In particular, secreted or surface-associated antigens might induce particularly strong cellular immune responses because of superior processing, kinetic advantages compared to internal antigens, and/or physical association with pathogen-associated molecular patterns (PAMP) such as lipopolysaccharide that provide potent stimuli for innate and adaptive immunity [14], [30]–[36]. Indeed, secretion/surface localization has been widely used to prioritize candidates for antigen identification. However, antigens with likely internal localization can also induce specific CD4 T cell responses that mediate protection against various intracellular pathogens [37], [38].

Taken together, relevant antigen properties for CD4 T cell mediated immunity to intracellular pathogens remain poorly characterized, and this impairs antigen priorization for vaccine development. To address this issue, we compared here 37 diverse *Salmonella* antigens in a mouse model that closely mimics human typhoid fever [39]. The results suggested that recognition of surface-associated antigens might be necessary to detect and combat live intracellular *Salmonella*, whereas recognition of internal antigens would mediate futile non-protective attack of already dead *Salmonella*. In conclusion, we propose a similar crucial role of surface-associated antigens for immunity to both extracellular and intracellular pathogens.

## Results

### Immune responses to *Salmonella* antigens in convalescent individuals

To determine immune responses to *Salmonella* antigens, we selected 21 broadly conserved *Salmonella* proteins. We selected several subunits of the SPI-2 type III secretion system since the putative translocon subunit SseB of this system showed promising protectivity in previous studies [12], [26]. We also included several porins since a previous study had shown that OmpD conferred protection against an attenuated *Salmonella* mutant [8]. To explore the role of antigen localization we selected additional proteins localized in *Salmonella* cytosol, inner membrane, periplasm, and outer membrane/surface. To explore the role of antigen abundance, we determined absolute quantities of more than 1100 *Salmonella* in infected mouse spleen. Specifically, we purified *Salmonella* from infected mouse spleen using flow cytometry as described [22]. We determined absolute protein quantities in these ex vivo purified *Salmonella* using shot-gun proteomics with 30 isotope labeled reference peptides and the iBAQ quantification method [40] (for detailed description see Materials and Methods section). From these data, we selected additional antigens with a large range of abundances (Tab. 1).

**Table 1:** Properties of *Salmonella* antigens.

STY antigen	STm orthologue	Name	Compartment	In vivo abundance in copies per cell ± SEM			CD154+ cells per 10^6^ CD4 T cells ± SEM			*P*-value^1^	IFNg-secreting cells per 10^6^ CD4 T cells ± SEM			*P*-value^1^	IL17-secreting cells per 10^6^ CD4 T cells ± SEM			*P*-value^1^	Serum IgG in ng ml^−1^ ± SEM			*P*-value^2^	Survival time extension in days	*P*-value^3^
T2461	STM0402		Cytosol	21725	±	2837	1949	±	733	0.008	591	±	251	0.014	268	±	82	0.005	3406	±	1852	<0.001	−3	0.924
T1689	STM1231	*phoP*	Cytosol	24060	±	1410	938	±	295	0.009	221	±	45	0.001	140	±	53	0.012	85	±	20	0.045	−1	0.702
T1265	STM1397	*sseA*	Cytosol	2163	±	877	703	±	245	0.030	137	±	17	0.001	84	±	20	0.002	36	±	7	0.310	−3	0.639
T0782	STM2090	*rfbH*	Cytosol	4260	±	392	1035	±	481	0.019	270	±	140	0.030	170	±	56	0.008	79	±	19	<0.001	−3	0.555
T0524	STM2340		Cytosol	922	±	434	923	±	520	0.038	276	±	119	0.020	103	±	30	0.009	36	±	0	<0.001	−1	0.782
T3053	STM3132		Cytosol	7504	±	1374	1083	±	618	0.030	322	±	98	0.009	198	±	71	0.016	52	±	10	<0.001	−2	0.676
T3595	STM4026	*yihX*	Cytosol	5101	±	902	1014	±	621	0.119	206	±	85	0.044	100	±	42	0.045	42	±	9	0.287	0	0.146
T1506	STM1597	*ydcW*	Cytosol	2328	±	462	952	±	652	0.378	139	±	66	0.030	106	±	65	0.022	46	±	9	0.006	3	0.268
T2763	STM2861	*sitA*	Inner Membrane	644	±	136	873	±	399	0.024	87	±	27	0.126	76	±	45	0.043	52	±	29	0.011	−4	0.938
T3872	STM3647	*yiaF*	Inner Membrane	829	±	354	n.d.				n.d.				n.d.				n.d.				0	0.880
T1508	STM1599	*pcgL*	Periplasm	14605	±	2135	503	±	235	0.077	75	±	22	0.006	63	±	66	0.110	1212	±	940	<0.001	−3	0.868
T0300	STM2556	*hmpA*	Periplasm	7658	±	1154	853	±	484	0.043	302	±	216	0.088	125	±	74	0.045	38	±	1	<0.001	−1	0.398
T4225	STM4319	*phoN*	Periplasm	37606	±	2742	559	±	263	0.021	155	±	39	0.004	59	±	30	0.038	2891	±	760	<0.001	−1	0.761
T2415	STM0445	*yajG*	Lipoprotein (outer membrane)	1057	±	360	n.d.				n.d.				n.d.				n.d.				−2	0.690
T1058	STM1819	*slp*	Lipoprotein (outer membrane)	599	±	208	n.d.				n.d.				n.d.				n.d.				−1	0.835
T3199	STM3281	*nlpL*	Lipoprotein (outer membrane)	b.t			n.d.				n.d.				n.d.				n.d.				−1	0.418
T0371	STM2488	*nlpB*	Lipoprotein (outer membrane)	2593	±	363	n.d.				n.d.				n.d.				n.d.				1	0.921
T0336	STM2520	*yfgL*	Lipoprotein (outer membrane)	2131	±	286	n.d.				n.d.				n.d.				n.d.				2	0.386
T2619	STM2663	*yfiO*	Lipoprotein (outer membrane)	763	±	289	n.d.				n.d.				n.d.				n.d.				4	0.441
T1459	STM1540		Lipoprotein (outer membrane)	5485	±	694	n.d.				n.d.				n.d.				n.d.				6	0.150
T3874	STM3645	*yiaD*	Lipoprotein (outer membrane)	3856	±	859	n.d.				n.d.				n.d.				n.d.				8	0.196
T0937	STM1940		Lipoprotein (outer membrane)	1864	±	396	546	±	650	0.128	67	±	89	0.140	49	±	25	0.013	37	±	2	<0.001	9	0.023
T1313	STM1445	*slyB*	Lipoprotein (outer membrane)	7270	±	719	n.d.				n.d.				n.d.				n.d.				12	0.228
T1262	STM1394	*ssaC*	Outer membrane	b.t			618	±	378	0.073	114	±	36	0.007	74	±	47	0.062	35	±	1	<0.001	−3	0.240
T3107	STM3186	*tolC*	Outer membrane	b.t			898	±	550	0.050	24	±	32	0.273	79	±	44	0.042	527	±	446	<0.001	−3	0.370
T0463	STM2395	*pgtE*	Outer membrane	8466	±	1032	1924	±	583	0.044	768	±	208	0.016	245	±	109	0.057	80	±	23	<0.001	−1	0.214
T1119	STM1246	*pagC*	Outer membrane	11993	±	3667	851	±	122	0.009	273	±	79	0.002	110	±	13	0.001	36	±	1	<0.001	−1	0.478
–	STM1572	*ompD*	Outer membrane	b.t			n.d.				n.d.				n.d.				n.d.				−1	0.958
T2450	STM0413	*tsx*	Outer membrane	6835	±	510	n.d.				n.d.				n.d.				n.d.				0	0.910
T0597	STM2267	*ompC*	Outer membrane	146	±	81	n.d.				n.d.				n.d.				n.d.				0	0.540
T0225	STM0224	*yaeT*	Outer membrane	1835	±	233	n.d.				n.d.				n.d.				n.d.				1	0.479
T1935	STM0999	*ompF*	Outer membrane	b.t			n.d.				n.d.				n.d.				n.d.				1	0.231
T2283	STM0585	*fepA*	Outer membrane	b.t			n.d.				n.d.				n.d.				n.d.				7	0.344
T2672	STM2777	*iroN*	Outer membrane	b.t			1038	±	569	0.037	353	±	166	0.023	155	±	58	0.013	41	±	6	0.180	16	0.012
T0656	STM2199	*cirA*	Outer membrane	b.t			n.d.				n.d.				n.d.				n.d.				16	0.139
T1266	STM1398	*sseB*	Secretion apparatus needle	n.a.			1048	±	304	0.016	329	±	143	0.012	104	±	28	0.006	410	±	170	<0.001	10	0.104
T1269	STM1401	*sseD*	Secretion apparatus translocon	n.a.			2177	±	410	0.003	823	±	357	0.050	381	±	125	0.031	202	±	85	<0.001	3	0.162

1 , t-test vs. non-infected control mice;

2 , t-test of log-transformed values vs. non-infected control mice;

3 , log-rank test vs. control mice immunized with GFP;

b.t.; below quantification threshold; n.d., not determined; n.a., not applicable for secreted proteins SseB and SseD.

To determine potential cross-protection between different serovars, we cloned the corresponding genes from *Salmonella enterica* serovar Typhi (except for OmpD which was obtained from serovar Typhimurium since it is absent in serovar Typhi). We expressed the proteins as C-terminal His_6_-fusions in *E. coli* followed by Ni-affinity chromatography purification. We purified the control antigen GFP-His_6_ using the same protocol.

**Figure 1:**
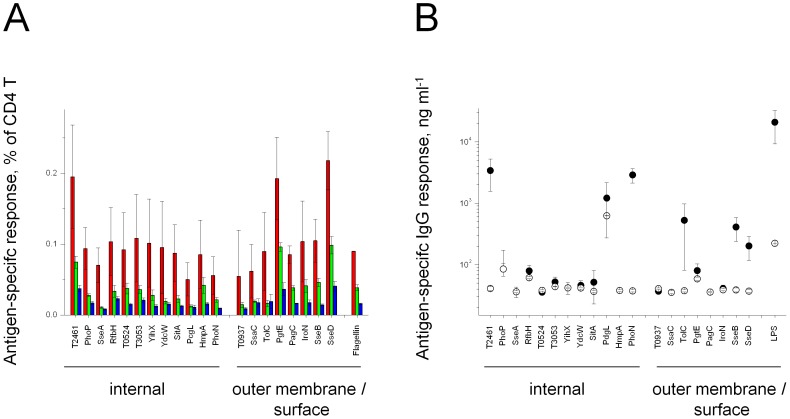
Cellular and humoral immune responses of convalescent *Salmonella*-infected mice to recombinant *Salmonella* antigens. **A**) Antigen-specific CD4 T cell frequencies as detected by CD154 upregulation (red) and IFNγ (green) or IL-17 (blue) secretion. The data represent means ± SE of three mice. Responses to *Salmonella* antigens in non-infected control mice were subtracted (see also Fig. S1). **B**) Serum antibody responses to *Salmonella* antigens. The data represent means ± SE of 11 convalescent mice (filled circles) and means ± SE for ten non-infected control mice (open circles).

We determined immune responses to these antigens in genetically resistant, convalescent mice that had survived infection with virulent *Salmonella enterica* serovar Typhimurium. We detected antigen-specific CD4 T cells in spleen using a sensitive CD154 assay [41] and measured serum IgG antibody responses using ELISA. All tested antigens were recognized by CD4 T cells (Fig. 1A; Tab. 1), many of which secreted IFNγ or IL-17 upon stimulation. Both cytokines play crucial roles in immunity to *Salmonella* [10]. Frequencies of responsive CD4 T cells were in the same range as for flagellin, which has been considered an immunodominant antigen [42]. These data suggested that *Salmonella* infection elicited a broad cellular immune response against a large number of in vivo expressed antigens from all *Salmonella* compartments in agreement with data observed for *S*. Typhi infected human patients [43]. There was no correlation between in vivo antigen abundance as determined by proteome analysis of ex vivo purified *Salmonella*, and CD4 T cell frequency or cytokine profile (Tab. 1).

Serum antibody responses revealed similar broad recognition of antigens from several *Salmonella* compartments (Fig. 1B) in agreement with previous data for human typhoid fever patients [24]–[26], [44]. Interestingly, the three immunodominant humoral antigens T2461, PhoN, and PcgL were all highly expressed in vivo (Tab. 1) suggesting a potential impact of antigen dose on antibody responses to *Salmonella*, although responses to minor antigens did not correlate with antigen abundance. PhoN has been previously recognized as an immunodominant antigen [26].

### Immunization and challenge infection

**Figure 2:**
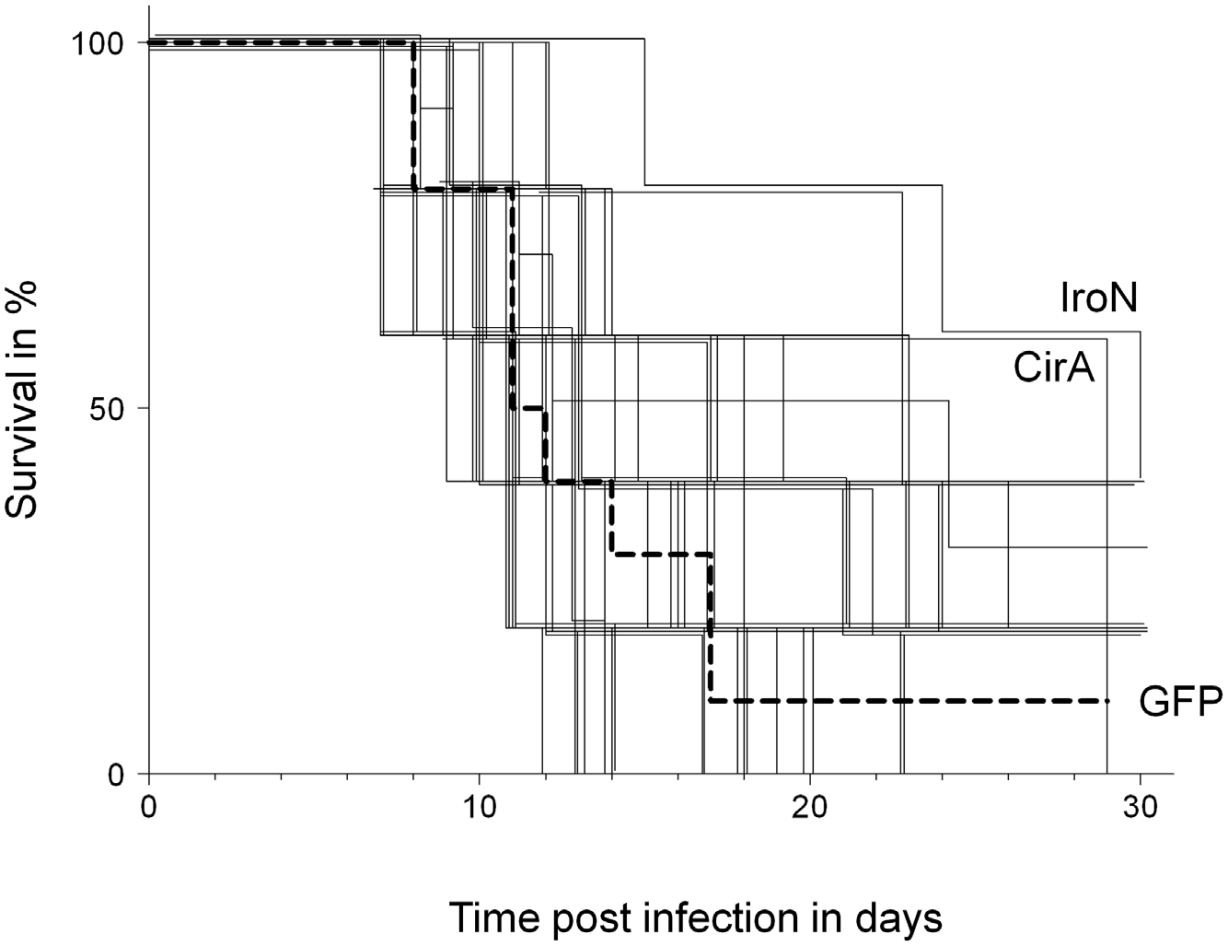
Survival curves of mouse groups immunized with 37 different *Salmonella* antigens (thin lines) or the control antigen GFP (thick dashed line). For better visualization, curves were slightly shifted. The longest survival was observed for antigens IroN and CirA. For statistical analysis by log-rank test see Table 1.

Many of the tested *Salmonella* antigens were capable to induce cellular and humoral immune responses. To test if these responses could confer protective immunity, we tested the 21 recombinant *Salmonella* antigens in immunization/challenge infection experiments in genetically susceptible BALB/c mice. Based on the results, we selected 16 additional *Salmonella* antigens primarily from the outer membrane, and tested them using the same experimental immunization/challenge approach (however, we did not measure their immunogenicity in convalescent mice). For simplicity, we discuss results for both antigen sets together. Out of 37 tested antigens, only few antigens enabled prolonged survival after oral challenge infection with virulent *Salmonella* compared to control immunization with the unrelated antigen GFP (Fig. 2; Tab. 1; poor survival of PhoN-vaccinated animals confirmed recently published data [26]). In fact, only two antigens (T0937 and T2672) mediated protective immune responses with *P*-values below 0.05 in our small experimental groups of only five mice per antigen. Replicate experiments with larger group sizes might yield statistical significant results for additional candidates such as SseB that has already been shown to be protective in two independent previous studies. Such experiments will be required to select individual antigens for vaccine development in future studies. On the other hand, the primary focus of this study was to identify antigen properties that correlate with protectivity. For this purpose, the somewhat noisy survival times detected with small animal groups were still helpful.

**Figure 3:**
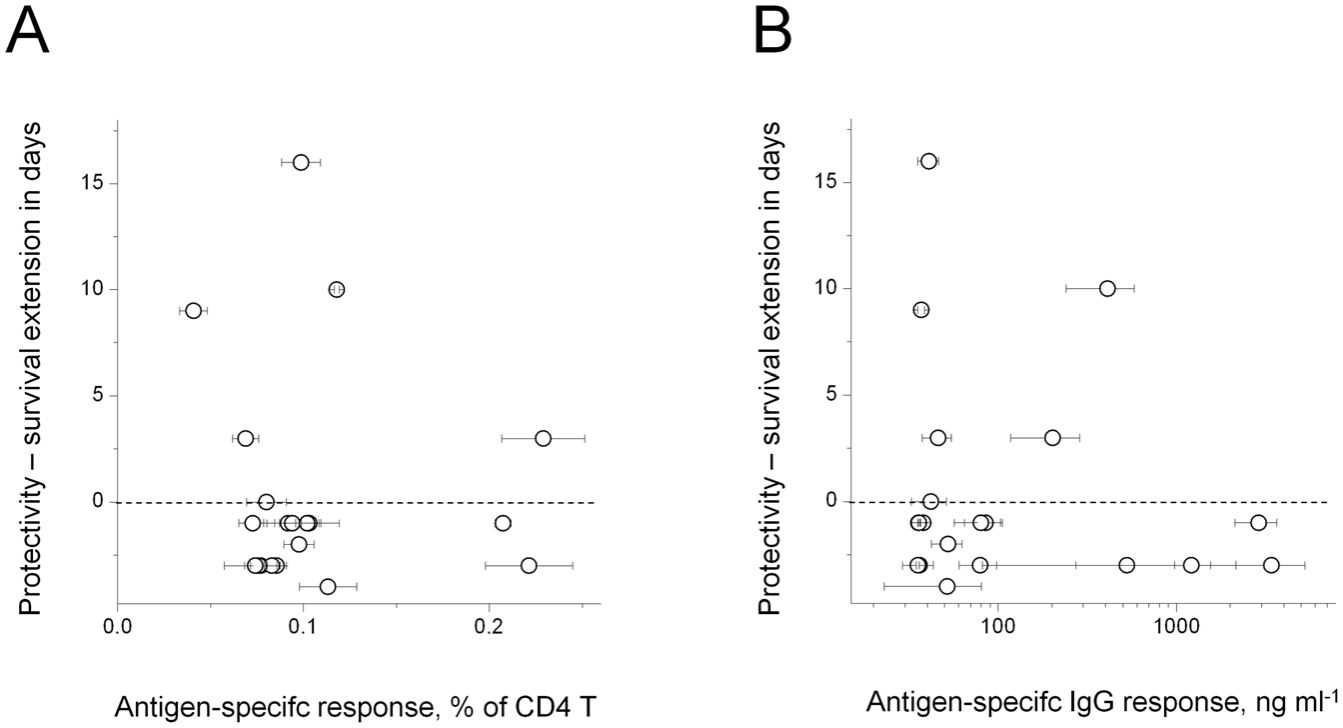
Comparison of *Salmonella* antigen protectivity against primary infection and immunogenicity in convalescent resistant mice. **A**) Relationship between antigen protectivity against primary infection and cognate CD4 T cell responses in convalescent mice (same data as Fig. 1A). Protectivity is expressed as “survival time extension”, which is the difference in median survival time of a group of five immunized mice compared to a control group of five mice that were immunized with GFP. **B**) Relationship between antigen protectivity and serum antibody responses in convalescent mice (same data as Fig. 1B).

As an example, survival times did not correlate with CD4 T cell responses (Fig. 3A) or serum antibody levels (Fig. 3B) during natural infection of resistant mice. This could partially reflect differences in MHC class II haplotypes (H2^d^ in BALB/c vs. H2^b^ in 129/Sv), courses of infection, and potential differences in *Salmonella* biology in susceptible vs. resistant mice. However, a recent large-scale study reports comprehensive immunogenicity data for BALB/c mice and other mouse strains that had been immunized with attenuated *Salmonella*, as well as for human patients [26]. Several antigens that prolonged survival of immunized BALB/c mice after *Salmonella* challenge infection in our experiments, elicit detectable antibody responses in various mouse strains including BALB/c. However, none of these antigens was found to be immunodominant [26] and antibodies to antigens IroN and CirA with the longest survival times were not detected in this and previous studies. This could reflect differential antigen expression in virulent vs. attenuated *Salmonella*, different routes of administration, and/or differential expression at various stages of disease progression. Together, these data provide no evidence for immunodominance in convalescent or immune individuals as a prerequisite for protectivity.

**Figure 4:**
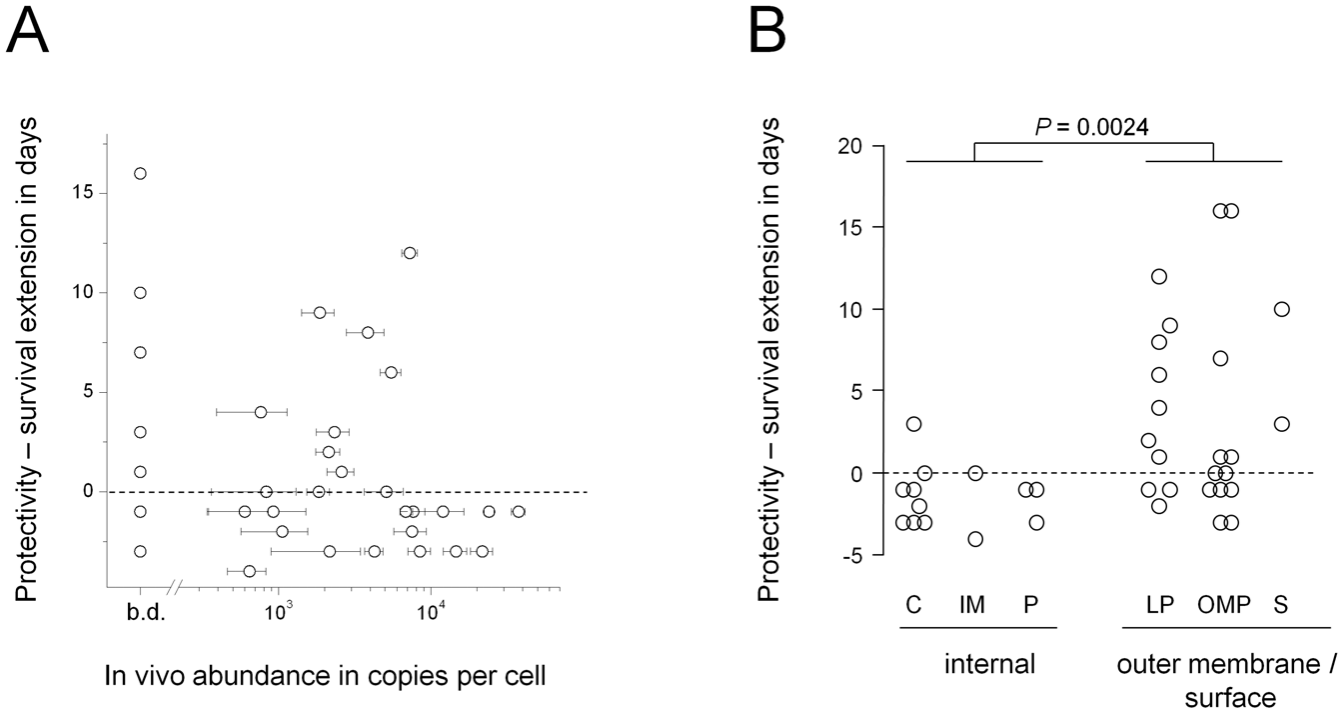
*Salmonella* antigen protectivity does not correlate with in vivo antigen abundance but depends on antigen localization within the *Salmonella* cell. **A**) Relationship between antigen protectivity and in vivo abundance as determined by quantitative proteome analysis of ex vivo purified *Salmonella* (means ± SD for three independently infected mice; b.d., below detection threshold). **B**) Relationship between antigen protectivity and antigen localization within *Salmonella* (C, cytosol; IM, inner membrane; P, periplasm; LP, outer-membrane associated lipoprotein; OMP, outer membrane protein; S, surface). Statistical significance of differences between internal and outer membrane/surface antigens was tested using the non-parametric Mann-Whitney U test.

Interestingly, in vivo expression levels also did not correlate with survival times (Fig. 4A). In fact, the two antigens that enabled the longest survival, IroN and CirA, had in vivo expression levels that were below our detection threshold. By comparison, antigens T2461 and PhoN were highly expressed in vivo and induced potent CD4 T cell and humoral responses in convalescent individuals, yet failed to prolong survival (in agreement with previous observations [26]).

In contrast to immunogenicity and in vivo abundance, antigen localization seemed to be crucial (Fig. 4B). In fact, antigens enabling prolonged survival times were exclusively associated with the *Salmonella* surface, either as experimentally validated outer membrane-associated lipoproteins [45], as outer membrane proteins, or as the translocon complex of the type III secretion system encoded by *Salmonella* pathogenicity island two (SPI-2) (Tab. 1). These data suggested distinct immune responses to *Salmonella* outer membrane/surface antigens that fundamentally differ from those to internal antigens.

**Figure 5:**
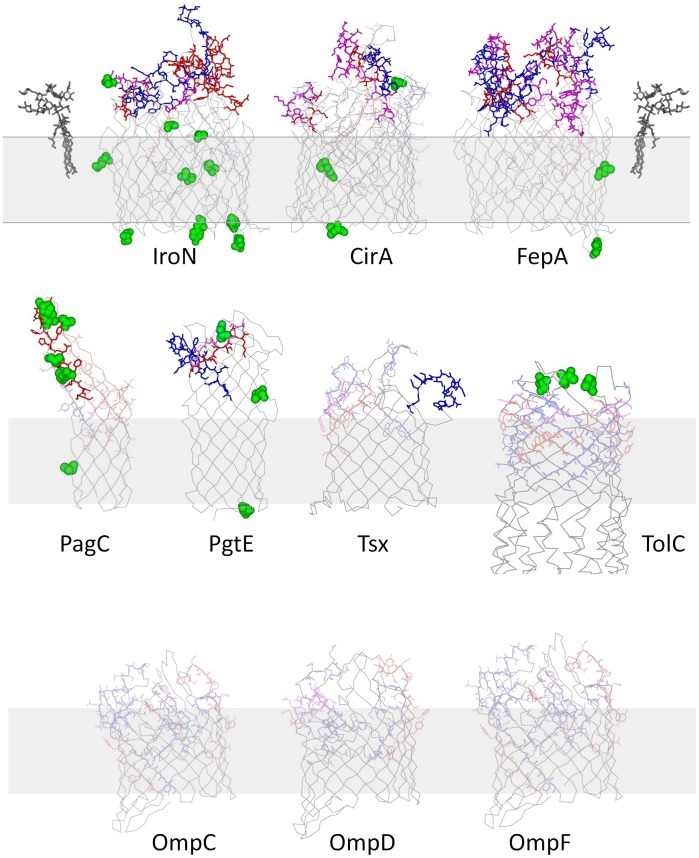
Structural models and exposed immune epitopes of various *Salmonella* outer membrane proteins. The outer membrane is shown as a grey area, predicted CD4 T cell epitopes in exposed loops are shown in red, potential antibody binding sites are shown in blue, and overlapping T and B cell epitopes are shown in magenta. Partially exposed epitopes are shown in pale colors. Amino acid residues that differ between *Salmonella enterica* serovars Typhimurium and Typhi are shown in green. For TolC only the outer membrane-associated part is shown. LPS structures as observed in FhuA-LPS crystals [93] are also shown.

On the other hand, surface localization alone was not sufficient for protectivity. As examples, membrane proteins PgtE, PagC, and Tsx were highly expressed in vivo and PgtE and PagC elicited potent CD4 T cell responses in convalescent individuals (Fig. 1A). PagC is also well recognized by antibodies and CD4 T cells of human typhoid fever patients [24]. However, PagC, PgtE, and Tsx failed to prolong survival. Interestingly, structural models revealed that these proteins were largely buried in the outer membrane bilayer (Fig. 5), and their extracellular loops contained at most one predicted CD4 T cell epitope each, and only up to two linear antibody epitopes, respectively. Importantly, key amino acids in exposed T cell epitopes differed among *Salmonella* serovars which might have impaired cross-protectivity of serovar Typhi antigens against serovar Typhimurium challenge infection. Similar observations were also made for non-protective TolC, OmpC, OmpD, and OmpF. By contrast, antigens IroN, CirA, and FepA that enabled extended survival after challenge infection, had extracellular loops with several highly conserved T and B cell epitopes (Fig. 5). Further studies with larger data sets will be required to validate the relevance of these structural properties for protectivity.

### Impact of *Salmonella* antigen localization in an ovalbumin model

The strong bias for surface-associated *Salmonella* antigens might have been expected based on previous data for model antigens suggesting superior immunogenicity of surface antigens compared to internal antigens [30], [46]–[50]. However, these model antigen data were in striking contrast to results from us and others demonstrating comparable immune responses to autologous *Salmonella* antigens from all *Salmonella* compartments (Fig. 1A,B). Furthermore, there was no obvious correlation between immunogenicity and survival times (Fig. 3A,B).

**Figure 6:**
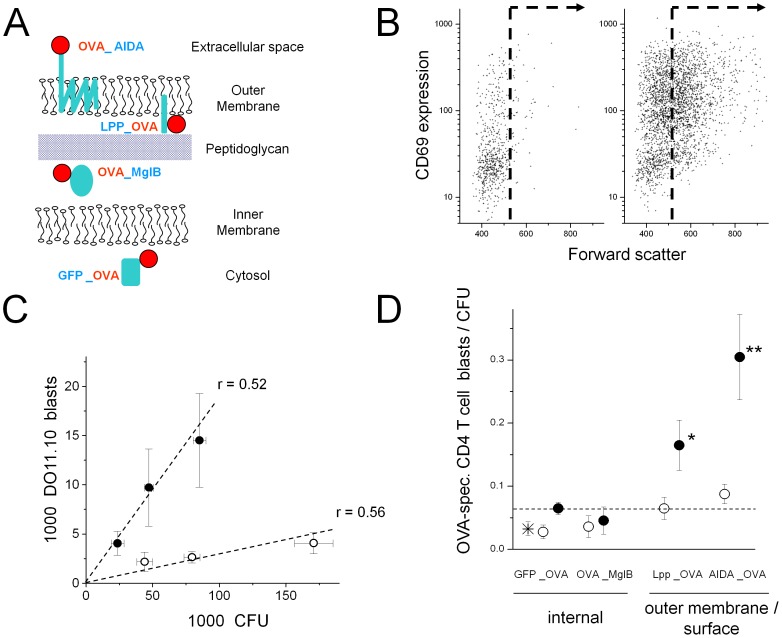
CD4 T cell responses to *Salmonella* expressing an ovalbumin model antigen in various compartments. **A**) Schematic overview of fusion proteins that target an immunodominant ovalbumin epitope (OVA) to various *Salmonella* cell compartments. **B**) Flow cytometric analysis of ovalbumin-specific CD4 T cell activation in a T cell receptor-transgenic adoptive transfer model. Mice were infected with control *Salmonella* expressing GFP (left) or *Salmonella* expressing LPP_OVA (right). Ovalbumin-specific transgenic CD4 T cells were detected with a clonotypic monoclonal antibody and analyzed for forward scatter and expression of the very early activation marker CD69. The dashed line was used to count CD4 T cell blasts. Similar observations were made for more than hundred mice in several independent experiments. **C**) Relationship between *Salmonella* Peyer's patches colonization and OVA-specific CD4 T cell induction in mice infected with *Salmonella* expressing high levels of LPP_OVA (filled circles) or low levels of GFP_OVA (open circles). Data represent means ± SEM's for groups of five to six animals from three independent experiments. CD4 T cell blasts correlated with *Salmonella* Peyer's patches colonization for both strains (Spearman test, *P*<0.05 in both cases). The slopes of the two curves differed (ANCOVA; *P*<0.05). **D**) OVA-specific CD4 T cell induction in mice infected with *Salmonella* expressing OVA at various levels (open circles, low abundance; filled circles, high abundance) in four different compartments. The dashed line represents CD4 T cell responses to saturating levels of cytosolic OVA. The star represents data for *Salmonella* expressing moderate levels of cytosolic OVA together with cholera toxin B and AIDA. Data represent means ± SEM's for groups of ten to twenty mice. Statistical significance of differences to *Salmonella* expressing saturating levels of cytosolic OVA were tested using Mann-Whitney U test (*, *P*<0.05; **, *P*<0.01).

To better understand these discrepancies between model antigens and autologous *Salmonella* antigens, we re-visited the impact of antigen localization using a well-characterized, sensitive model system in which a MHC II-restricted T cell epitope from ovalbumin comprising amino acids 319 to 343 (OVA) is recognized by adoptively transferred cognate T cell receptor transgenic CD4 T cells [51], [52]. We targeted the OVA epitope to different *Salmonella* compartments by fusing it to various proteins with known localization: GFP_OVA (cytosol [53]), OVA_MglB (periplasm [54]), Lpp_OVA (inner leaflet of the outer membrane [55]), and OVA_AIDA (outer leaflet of the outer membrane [56]) (Fig. 6A). To modulate expression levels, we used ribosome binding sites with differential translation initiation efficiency [12]. We expressed these fusion proteins from an in vivo inducible promoter [57] in an attenuated *Salmonella enterica* serovar Typhimurium *aroA* strain [58]. Antigen expression and localization was validated in in vitro cultures using cell fractionation followed by western blotting, trypsin treatment, and antibody binding (Fig. S2). Interestingly, small fractions of both outer membrane antigens LPP_OVA and partially processed OVA_AIDA were released to the extracellular surroundings when expressed at high levels (Fig. S2C) in agreement with previous findings for similar proteins [59]–[61].

We infected BALB/c mice with *Salmonella* strains by intragastric gavage of 10^10^ CFU. All *Salmonella* strains colonized intestinal Peyer's patches with peak tissue loads of 3×10^4^ to 1.5×10^5^ CFU at day seven post infection as observed before for attenuated *Salmonella aroA* [62]. All constructs stably maintained their respective ovalbumin-expression plasmids (>80% at 7 days post infection). To determine antigen-specific CD4 T cell induction, we adoptively transferred OVA-specific TCR-transgenic CD4 T cells one day prior to *Salmonella* infection. OVA-specific T cells upregulated the early activation marker CD69 and formed blasts in mice infected with *Salmonella* expressing ovalbumin model antigens, but not in mice infected with control *Salmonella* (Fig. 6B) as observed previously [53]. CD4 T cell induction kinetics were similar for all constructs and consistent with our previous observations [53] suggesting a response to *Salmonella* in situ antigen expression, but not to the inoculum [57], [63].

To compare T cell responses against the various *Salmonella* constructs, we measured T cell blast formation at peak *Salmonella* colonization at day seven post infection. *Salmonella* tissue loads varied somewhat between individual mice but for each construct, there was a linear relationship between the number of ovalbumin-specific DO11.10 blasts and *Salmonella* loads (Fig. 6C) in agreement with our earlier observations [57]. To determine the specific immunogenicity of each *Salmonella* strain, we calculated the average ratio of DO11.10 CD4 T cell blasts per viable *Salmonella* (i.e., the slopes in Fig. 6C) [57]. The data revealed comparable immunogenicity of model antigens GFP_OVA and OVA_MglB (Fig. 6D). In contrast, high-level expression of surface-associated LPP_OVA and OVA_AIDA induced superior responses that clearly surpassed responses even to saturating amounts [12] of internal GFP_OVA.

The OVA_AIDA fusion protein contained a fragment of the virulence factor AIDA from enteropathogenic *E. coli* and a cystein-deficient variant of the cholera toxin B subunit from *Vibrio cholerae* [64]. Both components might have stimulatory effects [65], [66] that could potentiate ovalbumin immunogenicity. To test this potentially confounding factor, we compared *Salmonella* expressing a suboptimal level of cytosolic GFP_OVA [12] (some 54.000 copies per *Salmonella* cell) to *Salmonella* expressing the same amount of GFP_OVA together with AIDA and cholera toxin B. Both strains induced DO11.10 T cell blasts with similar efficacy (Fig. 6D) suggesting that AIDA and cholera toxin B expression had no impact on the immunogenicity of *Salmonella*-encoded OVA.

Taken together, these findings suggested that antigens from all *Salmonella* compartments could induce specific CD4 T cell responses, but highly expressed outer membrane-associated antigens were clearly superior in agreement with previous observations in other model systems. However, these data were in striking contrast to responses to autologous *Salmonella* antigens (see discussion).

### Distribution of intact and damaged *Salmonella* in infected tissues

The fundamentally superior protectivity of surface-associated *Salmonella* antigens might reflect their unique accessibility to antigen processing and presentation in infected host cells in contrast to internal *Salmonella* antigens that are shielded by the *Salmonella* envelope, and thus remain invisible for the host immune system until *Salmonella* is damaged and the bacterial cell breaks open. To detect intact and damaged *Salmonella* in infected tissues, we used cytosolic GFP as a marker for internal antigens.

**Figure 7:**
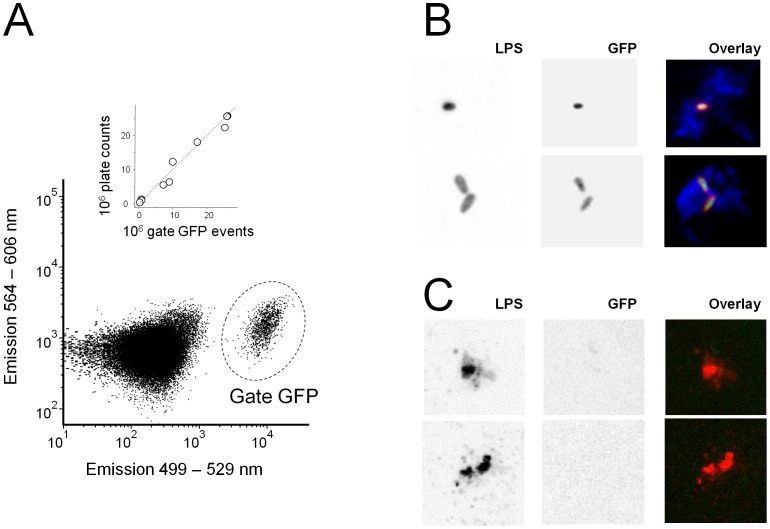
Detection of intact and damaged *Salmonella* cells in infected mouse tissues. **A**) Flow cytometry of a spleen homogenate infected with *Salmonella sifB::gfp* using 488 nm excitation. Gate 1 contains GFP-positive *Salmonella*. The inset shows the relationship between flow cytometry data and plate counts for individual mice, the dashed line represents a 1∶1 ratio. **B**) Confocal micrographs of liver cryosections infected with *Salmonella sifB::gfp* that were stained with antibodies to *Salmonella* lipopolysaccharide (red) and macrophage marker CD68 (blue). Individual color channels are shown with inverted grey scale for better visualization of weak staining. Micrographs represent typical observations for four independently infected mice. **C**) Confocal micrographs of lipopolysaccharide-positive particles that lack detectable GFP (even when contrast was increased compared to B). Such particles were absent in non-infected control sections.

*Salmonella* expressing GFP from the chromosomal in vivo induced locus *sifB* were readily detected in infected tissue homogenates using flow cytometry [12] (Fig. 7A). Flow cytometric counts for GFP^+^ *Salmonella* closely correlated with viable counts as determined by plating (Fig. 7A, inset) suggesting that detectable GFP levels were present in all live *Salmonella*.

Confocal microscopy of infected spleen and liver sections revealed many particles that were stained by a polyclonal antibody to *Salmonella* lipopolysaccharide, had typical *Salmonella* size and morphology, and contained GFP (Fig. 7B) as previously observed [53] suggesting that these particles represented live intact *Salmonella*. In addition, we also detected numerous lipopolysaccharide-positive particles with distorted shapes that lacked detectable GFP (Fig. 7C), and likely represented killed and partially degraded *Salmonella*. Such particles were absent in non-infected control sections. Some *Salmonella* killing during acute infections had previously been proposed [67]–[69]. We observed some infected cells containing both intact and damaged *Salmonella*, but a large number of live *Salmonella* resided alone (or together with other live *Salmonella*) in infected cells with no detectable dead *Salmonella*. In such infected cells, internal *Salmonella* antigens were thus shielded and inaccessible for immune recognition.

## Discussion

There is an urgent medical need for an efficacious *Salmonella* vaccine with broad coverage of invasive serovars. One important bottleneck in the development of such a vaccine is the identification of suitable protective antigens. In this study, we identified broadly conserved *S*. Typhi antigen candidates that prolonged survival after *S*. Typhimurium challenge infection in the mouse typhoid fever model. The protectivity of some of these candidates should be confirmed with larger experimental groups to select the best antigen candidates for vaccine development in future studies.

Two siderophore receptors (IroN, CirA) enabled the longest survival (Tab. 1) consistent with previous studies that revealed siderophore receptors including IroN as promising vaccine antigens in other models [70]–[72]. Interestingly, siderophore receptors are induced by iron starvation and/or activation of the PhoPQ two component sensory system [73]. IroN and CirA induction could thus contribute to increased protective efficacy of membrane preparations from iron-starved *Salmonella* [74], or live attenuated *Salmonella phoQ* ^24^ with constitutive hyperactivation of the PhoP response regulator [75].

On the other hand, all identified antigens still provided at most partial protection against challenge infection with virulent *Salmonella* suggesting a need for additional antigens. Unfortunately, protective *Salmonella* antigens might be rather rare as even among the 37 tested in vivo expressed antigens that were all highly immunogenic during infection, only a small minority enabled prolonged survival. OmpC, OmpD, and OmpF were previously proposed as potential protective antigens based on data obtained for enriched *Salmonella* membrane preparations. However, all three antigens failed to protect in our model. This could reflect higher stringency of our model (challenge infection with virulent *Salmonella* vs. highly attenuated mutant *Salmonella*), denatured three-dimensional structures of our recombinant antigen preparations vs. native antigens, and/or presence of undetected minor protective antigens (such as IroN and CirA) besides OmpC, OmpD, and OmpF in the previously used outer membrane antigen preparations.

Additional protective *Salmonella* antigens could be identified by comprehensive immunization/challenge experiments, but this would require extensive animal experimentation. Antigen priorization using relevant antigen properties could help to narrow down the number of antigen candidates to more practical numbers. Unfortunately, some previously proposed antigen properties seemed to have limited relevance for protectivity in our model. This included *Salmonella* in vivo expression levels, sequence-based antigenicity predictions, and immunodominance in convalescent individuals. Poor correlation of antigen immunodominance with protective efficacy has also been observed in tuberculosis [17]. On the other hand, immune recognition in convalescent individuals can still provide valuable information about antigen expression during at least some stage of infection that might be difficult to obtain otherwise [23], [29]. Such data thus could greatly help to prioritize antigen candidates [26].

In contrast to antigen abundance and immunodominance, surface-association appeared to be an essential prerequisite. Surprisingly, some surface-associated proteins that enabled prolonged survival also included lipoproteins which were likely to reside in the inner leaflet of the outer membrane facing the internal periplasmic space with no exposure to the outside. It is possible that some lipoproteins might flip across the outer membrane as observed for other Gram-negative bacteria [76]. Moreover, some lipoprotein fraction is constantly released to the outside through outer membrane vesicle shedding [59], [60].

Several mechanisms could contribute to the striking superiority of surface-associated antigens. Antibody responses are important for full protection against virulent *Salmonella* [10], and protective antibody responses must be directed against surface antigens [9]. On the other hand, CD4 T cells are even more important for immunity to *Salmonella* at least in the mouse typhoid fever model [10], and it is unclear why CD4 T cells should respond to surface-associated antigens in a fundamentally different way compared to the much larger number of internal antigens.

In fact, early cell culture experiments suggested no impact of *Salmonella* antigen localization on CD4 T cell recognition of infected cells [77]. However, in this study a large amount of antigen was already present in the inoculum, and rapid killing of the majority of phagocytosed *Salmonella* [78] would have released this antigen from all *Salmonella* compartments. Several subsequent in vivo studies suggested that surface-associated model antigens might have intrinsically higher immunogenicity compared to internal model antigens [30], [46]–[50]. However, the various model antigen targeting constructs could have differed in antigen in vivo expression levels, antigen stability, and epitope processing. Fusion partners could also have direct immunomodulatory effects. We therefore re-visited this issue and tried to control some of these factors. Our results clearly supported the previous finding of superior immunogenicity of highly expressed surface-associated model antigens in *Salmonella*.

In surprising contrast to these data from model antigens, however, humoral and cellular immune responses in *Salmonella*-infected convalescent mice did not show any bias for surface-associated autologous *Salmonella* antigens in this as well as in a recent large-scale study [26]. Broad recognition of antigens from all pathogens compartments has also been observed in *Salmonella* Typhi-infected or *Chlamydia*-infected human patients [24], [25], [29], [43], [44]. Model antigens and autologous antigens were also discordant with respect to the impact of antigen abundance. Specifically, our data for ovalbumin model antigens in this and a previous study [12], as well as similar findings for *Mycobacterium bovis* BCG overexpressing Ag85b [19], suggested that high in vivo expression levels enhance antigen immunogenicity. However, for autologous *Salmonella* antigens in vivo expression levels did not correlate with protectivity. Striking discrepancies between results for model antigens vs. autologous antigens have also been observed in other pathogens [38].

Some of the discrepancies could reflect technical issues. In particular, strong expression of foreign surface model antigens might induce subtle alteration in *Salmonella* in vivo properties such as increasing outer membrane vesicle shedding or alterations in protein secretion that could affect antigen presentation and immune recognition. Furthermore, model antigens might not be representative of autologous antigens that may have been shaped by host/pathogen co-evolution selecting for weak immunogenicity. Regardless of the actual causes of these discrepancies, our data indicated that in contrast to evidence from model antigens, protective *Salmonella* surface-associated antigens were not more immunogenic compared to internal antigens.

As an alternative explanation, surface-associated antigens might become more rapidly available for immune recognition compared to internal antigens that are only released after some pathogen damage. This could be relevant since early immune responses might facilitate infection control [32]. In the mouse typhoid fever model, however, a detectable fraction of *Salmonella* is rapidly killed early during infection as observed in this and previous studies [67], [69] similar to events during *Mycobacterium* infection [79]. Consistent with these observations, CD4 T cell induction kinetics in the ovalbumin model system were similar for *Salmonella* strains with internal or surface-associated OVA-expression.

**Figure 8:**
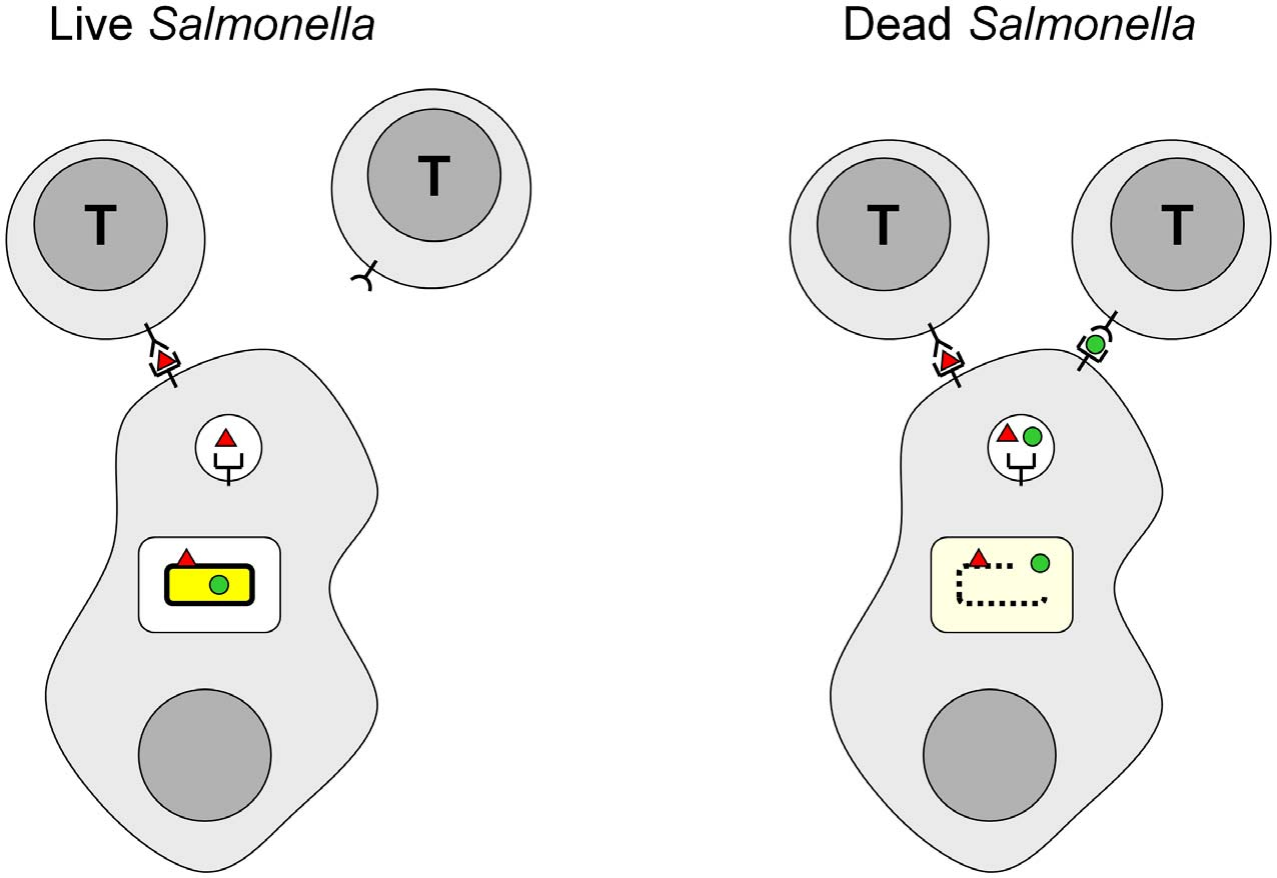
Schematic model for cellular immunity to *Salmonella*. *Salmonella* (yellow) reside in intracellular vacuoles in infected host cells. *Salmonella* possesses internal (green) and surface-associated (red) antigens. **Left**) Live *Salmonella* shield internal antigens, but some of their surface-associated antigens are accessible for processing and presentation. As a consequence, T cells specific for *Salmonella* surface antigens can recognize these infected cells and initiate antibacterial immune effector mechanisms. In contrast, T cells specific for internal *Salmonella* antigens fail to detect host cells that contain exclusively intact *Salmonella*. **Right**) Dead *Salmonella* release internal antigens. As a consequence, both surface-exposed and internal antigens can be processed, presented, and recognized by cognate T cells. However, this recognition is unproductive for infection control since it targets *Salmonella* that are already dead.

Instead, we propose an alternative explanation based on the observation that many live *Salmonella* resided alone, or together with other live *Salmonella*, in infected host cells with no dead *Salmonella* releasing their internal antigens. As a consequence, *Salmonella* internal antigens remained inaccessible for antigen processing and presentation in these cells. In contrast, surface-exposed *Salmonella* antigens, or antigens released by outer membrane vesicle shedding, could be accessible for processing and presentation to cognate CD4 T cells for initiation of protective anti-*Salmonella* effector mechanisms (Fig. 8). In comparison, CD4 T cells recognizing internal *Salmonella* antigens would have limited impact on infection control because they miss many cells containing live *Salmonella* and instead direct their responses to host cells containing already dead *Salmonella*. According to this model, surface-associated antigens thus differ fundamentally from internal antigens because they are uniquely accessible in host cells containing only live *Salmonella*.

Surface-associated/secreted antigens have been shown to be crucial for CD8 T cell-dependent immunity to *Listeria* infection [31], [33]. Our data suggested that such antigens might also be crucial for CD4 T cell mediated immunity to *Salmonella* and potentially other intracellular pathogens. Interestingly, some internal antigens have been shown to confer partial protection in infectious diseases caused by intracellular pathogens such as *Leishmania* [38] and *Mycobacterium* [37]. In these infections live and dead pathogens often co-occur in the same host microenvironments [80], [81] suggesting that both internal and surface-associated antigens might be available for T cell recognition and initiation of antimicrobial immune effector mechanisms targeting both live and already dead pathogens [82]. We speculate that full protection might still require immune detection of all live pathogens including those that reside in microenvironments with yet no accessible internal antigens from dead pathogens. Further studies are required to test this hypothesis.

### Conclusion

This study suggested novel *Salmonella* antigens that conferred partial protection against virulent *Salmonella* in a stringent typhoid fever model. High sequence conservation among relevant *Salmonella* serovars and cross-protection of serovar Typhi antigens against serovar Typhimurium challenge infection, suggested that some of these antigens might help to pave the way for a broadly protective vaccine against systemic *Salmonella* infection. In addition, our findings suggested that surface-associated antigens might represent particular promising antigens for both humoral and cellular immunity to *Salmonella*, since recognition of surface antigens uniquely enables detection and destruction of live *Salmonella* in relevant host microenvironments. This crucial importance of antigen localization could facilitate discovery of additional protective antigens for *Salmonella* and potentially other intracellular pathogens.

## Materials and Methods

### Ethics statement

All animal experiments were approved (license 2239, Kantonales Veterinäramt Basel-Stadt) and performed according to local guidelines (TschV, Basel) and the Swiss animal protection law (TschG).

### Cloning, expression, and purification of *Salmonella* antigens

Antigens were PCR-amplified from *Salmonella enterica* serovar Typhi Ty2 (or *Salmonella enterica* serovar Typhimurium SL1344 [58] for *ompD*), cloned as His_6_-fusions by conventional ligation into pET22b, or by Enzyme Free Cloning into plasmid pLICHIS [83], and overexpressed in *E. coli* BL21. GFP_His_6_ was cloned as control antigen. Antigens were purified from washed inclusion bodies using immobilized metal ion affinity chromatography (Protino Ni TED 1000, Macherey Nagel) followed by ion exchange chromatography (Ion exchange spin columns, Pierce Thermo Scientific, cationic or anionic resins depending on antigen isoelectric point).

### Proteome analysis of ex vivo sorted *Salmonella*

*Salmonella* expressing the green fluorescent protein (GFP) were sorted infected using flow cytometry as described [22]. Preparation of tryptic peptides and analysis by LC-MS/MS was done essentially as described [84] with some modifications. Given the limited sample material Protein LoBind tubes and pipette tips (Axygen) were used throughout the procedure. Frozen FACS sorted *Salmonella* pellets were resuspended in 15 µl lysis buffer (100 mM ammonium bicarbonate, 8 M urea, 0.1% RapiGest) and sonicated for 2×30 seconds. The released proteins were reduced and alkylated, and first digested for 4 hrs with sequencing grade LysC peptidase (10 ng/µl; Promega) before overnight trypsin digestion. The detergent was cleaved by adding 2M HCL and 5% TFA to final concentrations of 50 mM and 0.5% respectively, and incubating for 45 min at 37°C. Prior to centrifugation to remove the cleaved detergent (14,000×g, 10 min, 4°C) a mixture containing 32 heavy labeled reference peptides were added to the samples (5*10^−5^ fmoles per *Salmonella* for expected “high” abundance proteins, 5*10^−6^ fmoles per *Salmonella* for expected “low” abundance proteins; Tab. S1). The recovered peptides were desalted on C18 reverse-phase spin columns (Macrospin columns, Harvard apparatus), dried under vacuum and subjected to LC-MS/MS using an LTQ-Orbitrap-Velos instrument (Thermo-Fischer Scientific). The amount of material analyzed in a single shot in the MS depended on the infection load, and corresponded to peptides derived from between 5*10^5^ and 2*10^6^ sorted *Salmonella*, plus contaminating mouse material which escaped detection in the cell sorter [22]. We analyzed samples from seven independently infected mice. In order to increase the number of *Salmonella* protein identifications, MS-sequencing was focused on previously identified peptides from *Salmonella* using the recently developed inclusion list driven workflow [84]. Each sample was analyzed twice in succession in the MS to verify technical reproducibility. Peptides and proteins were database searched against a decoy database consisting of the SL1344 genome sequence (ftp://ftp.sanger.ac.uk/pub/pathogens/Salmonella/), GFP_OVA, 204 frequently observed contaminants, all mouse entries from SwissProt (Version 57.12), and all sequences in reversed order (total 42502 entries) using the Mascot search algorithm. The search criteria were set as follows: full tryptic specificity was required (cleavage after lysine or arginine residues); 2 missed cleavages were allowed; carbamidomethylation (C) was set as fixed modification; oxidation (M) as variable modification. The mass tolerance was set to 10 ppm for precursor ions and 0.5 Da for fragment ions. The false discovery rate was set to 1% for protein and peptide identifications. In addition to *Salmonella* proteins a substantial number of mouse proteins were identified in the samples as previously noted [22]. Absolute quantities were determined for those 18–20 “anchor” proteins that were detected along with a corresponding labeled AQUA peptide using the Trans-Proteomic Pipeline (TPP,V4.4.0). We then used the iBAQ method [40] to establish absolute quantities of all remaining protein identifications, with a linear model error of between 47 and 60%.

### Construction of ovalbumin-expressing *Salmonella*

Translational fusions of the ovalbumin peptide containing amino acids 319 to 343 to various proteins with differential targeting in the *Salmonella* cell were constructed by PCR cloning. All fusion genes were cloned into a pBR322-derived plasmid backbone [53] downstream of a *Salmonella* genome fragment containing the in vivo inducible *pagC* promoter [57] and ribosomal binding site 1 (AAGAA) or 2 (AGCAG) for low or high translation initiation efficiencies [12]. To generate *ova_aida*, coding sequence for the ovalbumin peptide (*ova*) was inserted between the signal peptide derived from cholera toxin B and the HA tag in plasmid pLAT260 [85]. A control plasmid coding for CTB_AIDA and GFP_OVA was also constructed. To generate *lpp_ova*, *lpp* without the C-terminal lysine codon that can cross-link to peptidoglycan [86], was amplified from *E. coli* DH5α and fused with *ova* and a C-terminal HA tag. To generate *ova_mglB*, *mglB* gene without the signal peptide sequence was amplified from *E. coli* DH5α and fused with a *ctB* signal sequence followed by *ova* and the HA tag. The construction of *gfp_ova* has been described [87]. The various plasmids were transformed into attenuated *Salmonella enterica* serovar Typhimurium aroA SL3261 [58].

### Biochemical analysis

Ovalbumin expression was assessed by western blotting with a polyclonal antibody to ovalbumin (Sigma) that recognizes the OVA peptide comprising amino acids 319 to 343 [53]. *Salmonella* outer membranes were prepared by extraction with L-lauryl sarcosinate as described [85]. Periplasm was prepared by chloroform extraction as described [88]. Culture supernatants were sterile filtered (0,2 µm pore size) and subjected to TCA precipitation [89]. To assess ovalbumin surface accessibility, intact or lysed *Salmonella* cells were treated with 50 µg ml^−1^ trypsin at 37°C for 10 min. In addition, *Salmonella* were stained with an antibody to the HA tag, and examined by fluorescence microscopy.

### Immune responses in convalescent mice

Female 8 to 12 weeks old 129/Sv mice were obtained from Charles River. Mice were orally infected with 10^9^ CFU *Salmonella enterica* serovar Typhimurium SL1344 [58] from late log cultures using a round-tip stainless steel needle. Control mice were sham-infected. Mice were sacrificed 6 months after infection. Splenocytes were isolated and tested for antigen-specific CD4 T cell responses as described [41]. Unstimulated T cells from convalescent mice as well as antigen-stimulated T cells from naïve control mice showed only weak background responses (Fig. S1). Some antigens gave also weak responses for T cells from convalescent mice (depending on the individual mouse). Together, these data suggested that antigen-nonspecific background responses to *E. coli* contaminants that might have been present in trace amounts in our antigen preparations did not result in unspecific T cell responses in our assay. Plasma was tested for antigen-specific IgG responses using ELISA with an IgG calibration curve for absolute quantification.

### Immunization and challenge experiments

Female, 8 to 12 weeks old BALB/c mice were obtained from Charles River. Groups of 5 mice were immunized subcutaneously with 10 µg antigen emulsified in complete Freund's adjuvant followed by a second immunization with incomplete Freund's adjuvant four weeks later. After additional four weeks, mice were orally infected with 6×10^5^ CFU *Salmonella enterica* serovar Typhimurium SL1344 [58] from late log cultures using a round-tip stainless steel needle. Infected BALB/c were monitored twice daily and sacrificed when moribund.

### Ovalbumin-specific CD4 T cell responses

BALB/c and DO11.10 mice [51] were bred in the Bundesamt für gesundheitlichen Verbraucherschutz und Veterinärmedizin (Berlin, Germany) under specific-pathogen free conditions. Adoptive transfer of 4×10^6^ DO11.10 T cells into syngenic age- and sex-matched BALB/c mice was performed one day before infection as described [53]. For infection, attenuated *Salmonella* strains carrying expression cassettes for various ovalbumin fusion proteins were grown to late log phase and harvested. Bacteria were washed twice and resuspended in LB containing 3% sodium bicarbonate. Doses containing ca. 10^10^ cfu in 100 µl were administered intragastrically to chimeric mice with a round-tip stainless steel needle. At various time points post infection, mice were anesthetized and sacrificed. DO11.10 T cell blast formation was determined by flow cytometry as described [53]. Aliquots of the same Peyer's patch preparations were treated with 0.1% Triton x-100 to release intracellular *Salmonella* for CFU determination by plating, and for quantitation of GFP_OVA *in vivo* expression levels by two-color flow cytometry as described [87]. Many TCR tg models show substantial clonal expansion upon antigen stimulation. However, in our *Salmonella* model we observe only weak and variable accumulation of tg CD4 T cells in infected tissues which might reflect the fact that even at peak *Salmonella* loads only about 1 ng antigen is present [87]. Instead, blastogenesis as measured by CD69 upregulation and increased forward scatter provides a sensitive antigen-specific readout.

### Detection of intact *Salmonella* in infected tissues

BALB/c mice with *Salmonella* loads of 10^6^ to 10^7^ in spleen and liver were sacrificed. 10 µm cryosections were stained with polyclonal rabbit antibodies to *Salmonella* lipopolysaccharide (SIFIN) and anti-CD68 (abcam) followed by Alexa 546-conjugated goat anti-rabbit and Alexa 647-conjugated goat anti-rat antibodies (Invitrogen). Sections were examined by confocal microscopy (Leica, SP5).

### Structural models and epitope prediction

Structural models for selected *Salmonella* outer membrane antigens based on solved structures of homologues were obtained from SWISS-MODEL [90] available at http://swissmodel.expasy.org. Linear B-cell epitopes were predicted using FBCPred [91] available at http://ailab.cs.iastate.edu/bcpreds/predict.html using an epitope length of 14 and 90% specificity. Peptides that bind to MHC II I-A^d^ and/or I-E^d^ were predicted using RANKPEP [92] available at http://imed.med.ucm.es/Tools/rankpep.html with a binding threshold yielding 85% sensitivity for detection of well-defined epitopes in MHCII haplotype databases (the default setting of RANKPEP).

## Supporting Information

Figure S1 **Representative antigen-specific CD4 T cell responses in convalescent and control mice.** Cells were stimulated with various antigens (shown are examples for T2461 and T0937). CD4, CD154-double positive cells representing responding cells were gated (upper panels) and analyzed for IFNγ and IL-17 (lower panels). Background responses in control mice were subtracted form responses in convalescent mice and reported in Tab. 1.
(TIF)

Figure S2 **Expression and localization of ovalbumin epitope fusion proteins in** ***Salmonella*** **.** **A**) Anti-ovalbumin immunoblot of total *Salmonella* cell lysates (3×10^7^ cfu) of strains expressing either low (“lo”) or high (“hi”) levels of ovalbumin fused to different proteins. Expected molecular weights were: GFP_OVA, 30 kDa; OVA_MglB, 38 kDa; Lpp_OVA, 11 kDa; OVA_AIDA, 67 kDa. **B**) Localization of various fusion proteins. OVA_MglB was detected in isolated periplasm fractions (Ppl.) in similar quantities as in whole cell lysates (Lys.). Lpp_OVA was detected in isolated outer membrane fractions. It was unaccessible for trypsin degradation in intact *Salmonella* but readily digestible in isolated membrane fractions. Immunostaining of intact *Salmonella* with a fluorescent antibody showed no detectable signal. OVA_AIDA was detected in isolated outer membranes and accessible to trypsin digestion even in intact *Salmonella* suggesting surface localization. This was confirmed by immunostaining. **C**) Immunoblot of culture supernatants of 4.5×10^11^ CFU (TCA precipitation). Endogenous *Salmonella* proteins with apparent molecular weights of ca. 23 and 67 kDa, respectively, cross-react with the anti-ovalbumin polyclonal antibody (empty arrowheads). These bands were also detected in non-recombinant *Salmonella*. In addition, an OVA-containing protein of around 11 kDa was released from Lpp_OVA expressing *Salmonella* (black arrowhead), whereas a 30 kDa fragment was released from *Salmonella* expressing high amounts of OVA_AIDA.
(TIF)

Table S1 **List of isotope labeled peptides used for protein quantification.**
(XLS)
