# Supplementary figures and images for: Immunity to Intracellular Salmonella Depends on Surface-associated Antigens
Source: PLoS Pathog. 2012 Oct 18;8(10):e1002966. doi: 10.1371/journal.ppat.1002966 (PMC3475680; doi:10.1371/journal.ppat.1002966)

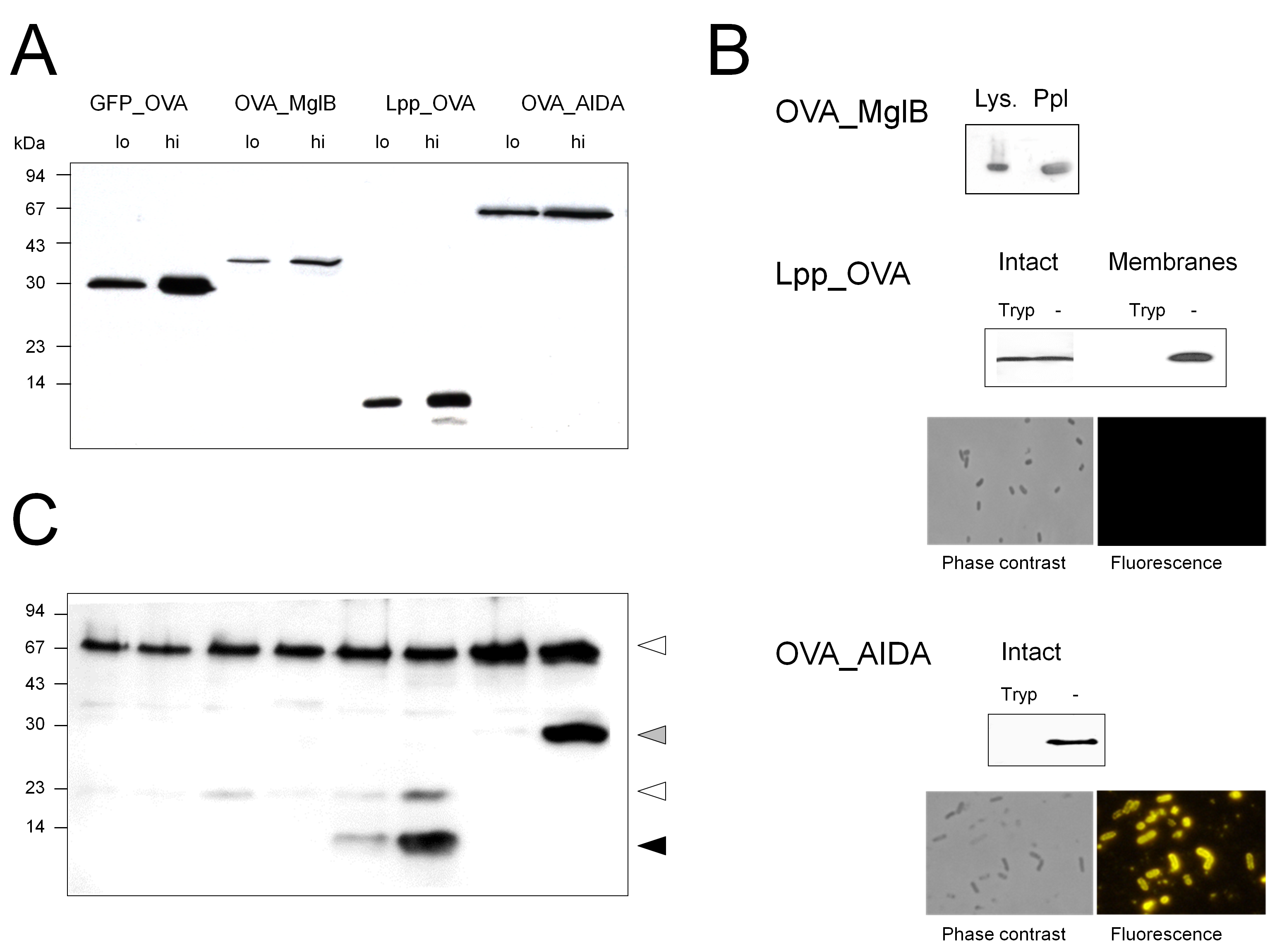

Supplement: Figure S2 — Expression and localization of ovalbumin epitope fusion proteins in Salmonella . A) Anti-ovalbumin immunoblot of total Salmonella cell lysates (3×10^7^ cfu) of strains expressing either low (“lo”) or high (“hi”) levels of ovalbumin fused to different proteins. Expected molecular weights were: GFP_OVA, 30 kDa; OVA_MglB, 38 kDa; Lpp_OVA, 11 kDa; OVA_AIDA, 67 kDa. B) Localization of various fusion proteins. OVA_MglB was detected in isolated periplasm fractions (Ppl.) in similar quantities as in whole cell lysates (Lys.). Lpp_OVA was detected in isolated outer membrane fractions. It was unaccessible for trypsin degradation in intact Salmonella but readily digestible in isolated membrane fractions. Immunostaining of intact Salmonella with a fluorescent antibody showed no detectable signal. OVA_AIDA was detected in isolated outer membranes and accessible to trypsin digestion even in intact Salmonella suggesting surface localization. This was confirmed by immunostaining. C) Immunoblot of culture supernatants of 4.5×10^11^ CFU (TCA precipitation). Endogenous Salmonella proteins with apparent molecular weights of ca. 23 and 67 kDa, respectively, cross-react with the anti-ovalbumin polyclonal antibody (empty arrowheads). These bands were also detected in non-recombinant Salmonella. In addition, an OVA-containing protein of around 11 kDa was released from Lpp_OVA expressing Salmonella (black arrowhead), whereas a 30 kDa fragment was released from Salmonella expressing high amounts of OVA_AIDA. (TIF) [file ppat.1002966.s002.tif]
